# The new visceral adiposity index outperforms traditional obesity indices as a predictor of subclinical renal damage in Chinese individuals: a cross-sectional study

**DOI:** 10.1186/s12902-023-01330-5

**Published:** 2023-04-07

**Authors:** Yue Sun, Yu Yan, Yueyuan Liao, Chao Chu, Tongshuai Guo, Qiong Ma, Yang Wang, Dan Wang, Hao Jia, Jianjun Mu

**Affiliations:** 1 Department of Cardiology, First Affiliated Hospital of Medical School, Xi’an Jiaotong University, No.277, Yanta West Road, Xi’an, 710061 Shaanxi China; 2 Key Laboratory of Molecular Cardiology of Shaanxi Province, Xi’an, China; 3 Key Laboratory of Environment and Genes Related to Diseases (Xi’an Jiaotong University), Ministry of Education, Xi’an, Shaanxi, China

**Keywords:** Chronic kidney disease, Subclinical renal damage, Visceral adiposity

## Abstract

**Background:**

The new visceral adiposity index (NVAI) was superior to previous obesity indices in predicting cardiovascular diseases among Asians. Nevertheless, the utility of the NVAI for predicting chronic kidney disease is still unclear. The objective of this research was to explore the relationship between the NVAI and subclinical renal damage (SRD) and to investigate whether the NVAI outperforms other common obesity indices in predicting SRD in the Chinese population.

**Methods:**

Participants in this cross-sectional study were from the Hanzhong Adolescent Hypertension Cohort. The NVAI and seven other common obesity indices were calculated, including body mass index, waist circumference, lipid accumulation product, visceral adiposity index, Chinese visceral adiposity index, a body shape index and metabolic score for visceral fat. Logistic regression models revealed the association between NVAI and SRD. The odds ratio (OR) and the 95% confidence interval (CI) were calculated to show the association between the two variables. The predictive power of eight obesity indices for SRD was evaluated through the receiver operating characteristic curve and area under the curve (AUC). In addition, the net reclassification index (NRI) and integrated discrimination improvement (IDI) were also applied to compare the incremental predictive value for SRD of different obesity indices.

**Results:**

The median age of the 2358 subjects was 42.00 years. Across NVAI tertiles, the prevalence of SRD was 7.25%, 11.21%, and 21.60%, respectively. After adjusting for confounders, a high level of NVAI remained a risk factor for SRD. The ORs of the middle and top NVAI tertiles for SRD were 1.920 (95% CI: 1.322, 2.787) and 4.129 (95% CI: 2.750, 6.202), respectively. The AUC of the NVAI was 0.666 (95% CI: 0.647, 0.685), which was significantly larger than the AUC of any of the other obesity indicators. Moreover, the NRI and IDI were significantly improved when NVAI was added to the basic model for predicting SRD. Among eight obesity indices, NVAI had the highest NRI (0.392; 95% CI: 0.280, 0.503), and its IDI (0.021; 95% CI: 0.014, 0.027) was second only to that of the body mass index (0.023; 95% CI: 0.014, 0.032).

**Conclusions:**

NVAI is independently and positively associated with SRD. Among the eight obesity indices, the NVAI shows the strongest predictive power for SRD in the Chinese population. The NVAI may be useful as an effective warning indicator of chronic kidney disease in Chinese adults.

**Supplementary Information:**

The online version contains supplementary material available at 10.1186/s12902-023-01330-5.

## Introduction

Chronic kidney disease (CKD) has become a global health issue [1]. The prevalence and mortality of CKD have increased dramatically since 1990 [2]. In 2017, almost 700 million patients struggled with CKD worldwide, with more than 1 million cases dying as a result [2]. Defined as the continuously abnormal structure or function of the kidney for more than three months [3], CKD consists of different stages based on the level of albuminuria or the glomerular filtration rate [2, 4]. Once in end-stage kidney disease, which means poor renal function with no chance of recovery, the quality of life of subjects is always compromised by a slew of negative symptoms [5, 6]. Therefore, it is necessary to identify and treat subclinical renal damage (SRD) as early as possible.

Obesity greatly contributes to the occurrence and deterioration of CKD [7, 8]. According to previous research, the abundance of visceral fat rather than overall obesity is a crucial factor in the link between obesity and CKD [9, 10]. Body mass index (BMI) is frequently utilized in medical practice to measure obesity, but it is unable to reflect regional fat distribution, especially in CKD patients [11, 12]. However, accurate detection methods such as magnetic resonance imaging or computed tomography are not only costly but may be harmful because of exposure to radiation [13]. Therefore, alternative parameters have been developed, including the visceral adiposity index (VAI) [14], Chinese VAI (CVAI) [15], lipid accumulation product (LAP) [16], a body shape index (ABSI) [17], and metabolic score for visceral fat (METS-VF) [18], which have been shown to be related to CKD [19–23]. Recently, it was documented that a new VAI (NVAI) had a higher predictive value for cardiovascular diseases than other obesity indicators in the Korean population [24, 25]. However, research exploring any association between the NVAI and SRD is still absent.

Therefore, this study aimed to investigate among Chinese adults whether the NVAI is a predictor of SRD and compare its predictive value with other obesity indices.

## Methods

### Study participants

**Fig. 1:**
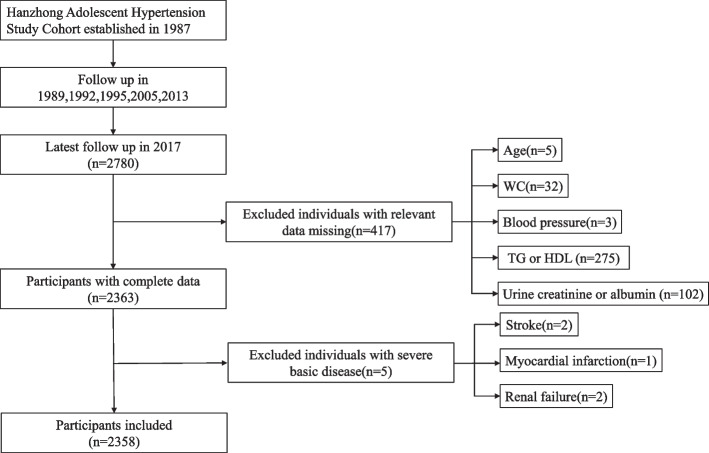
Flow diagram showing the recruitment of participants in the cross-sectional study. Legends of Fig. 1: HDL, high-density lipoprotein; TG, triglyceride; WC, waist circumference

The Hanzhong Adolescent Hypertension Cohort is a currently underway prospective study with the goal of exploring the development of cardiovascular risk factors since childhood. It was established in 1987, and so far, it has been followed up six times for 30 years. Details of the cohort were published previously [26]. The latest follow-up was in 2017, involving 2780 subjects. A total of 417 individuals without relevant data for NVAI or renal function indicators were excluded. After further excluding five patients with severe basic disease, the cross-sectional study eventually included 2358 subjects (Fig. 1).

The research adhered to the guidelines of the Helsinki Declaration. The Ethics Committee of the First Affiliated Hospital of Xi’an Jiaotong University approved the current study. Informed consent forms were signed by all participants.

### Anthropometric measurements

Uniformly trained staff obtained detailed demographic information, history of diseases, smoking habits, and alcohol consumption of the subjects through a standard questionnaire. Participants were measured for waist circumference (WC), height, and weight while wearing only underwear and no shoes. The mean of two measurement values of height was included in the statistical analysis, as was weight. In a quiet environment, a professional used a precalibrated sphygmomanometer to measure the blood pressure of the individuals in a sitting position. Three measurements were conducted at 2-min intervals. The mean was taken as the actual blood pressure level. The value combining 1/3 systolic blood pressure (SBP) with 2/3 diastolic blood pressure (DBP) was determined as the mean arterial pressure (MAP).

Hypertension was defined as SBP ≥ 140 mmHg, DBP ≥ 90 mmHg, taking antihypertensive drugs, or a self-reported history of hypertension. Diabetes was defined as fasting plasma glucose (FPG) ≥ 7.0 mmol/l, taking hypoglycemic medications, or a self-reported history of diabetes.

### Biochemical assays

Venous blood samples of individuals who had fasted for at least eight hours were collected by professional staff in the morning. Detection of lipids, renal function, and liver function was subsequently conducted. Relevant biochemical parameters consisted of high-density lipoprotein (HDL), low-density lipoprotein (LDL), triglyceride (TG), total cholesterol (TC), uric acid (UA), creatinine, glutamic pyruvic transaminase (GPT), glutamic oxaloacetic transaminase (GOT), total bile acid (TBA), and FPG. Using spot urine samples, the physicians measured the concentrations of creatinine, albumin, and UA with an automated device.

### Obesity indicators

A series of obesity indices were calculated using the following equations [14–18, 24].

1 $$\mathrm{BMI}\text{=}\frac{\text{body weight}}{{\text{height}}^{2}}$$

2 $$\mathrm{ABSI}=\frac{\mathrm{WC}}{{\mathrm{BMI}}^{2/3}\times {\mathrm{height}}^{1/2}}$$

3 $$\mathrm{WHtR}=\frac{\mathrm{WC}}{\mathrm{height}}$$

4 $$\mathrm{METS}-\mathrm{IR}=\frac{\mathrm{Ln}[\left(2\times \mathrm{FPG}\right)+\mathrm{TG}]\times \mathrm{BMI}}{\mathrm{Ln}(\mathrm{HDL})}$$

For men:

5 $$\text{NVAI }\text{=}\frac{1}{\text{1} + {\text{e}}^{-\left[\text{-21.858+}\left(\text{0.099}\times {\text{age}}\right)\text{+}\left(\text{0.10}\times {\text{WC}}\right)\text{+}\left(\text{0.12}\times {\text{MAP}}\right)\text{+}\left(\text{0.006}\times {\text{TG}}\right)\text{+}\left(\text{-0.077}\times {\text{HDL}}\right)\right]}}$$

6 $$\text{CVAI } = \text{-267.93+}\left(\text{0.68}\times {\text{age}}\right)\text{+}\left(\text{0.03}\times {\text{BMI}}\right)\text{+}\left(\text{4.00}\times {\text{WC}}\right)\text{+}\left({22}\times {\text{log}}{\text{TG}}\right)-\left(\text{16.32}\times {\text{HDL}}\right)$$

7 $$\text{VAI }\text{=}\frac{\text{WC}}{\text{39.68+}\left(\text{1.88}\times {\text{BMI}}\right)}\times \frac{\text{TG}}{1.03}\times \frac{1.31}{\text{HDL}}$$

8 $$\text{LAP } = \left(\text{WC-65}\right)\times {\text{TG}}$$

9 $$\mathrm{METS}-\mathrm{VF}=4.466\text{+0.011}\times \text{[Ln(}{\mathrm{METS}-\mathrm{IR})]}^{3}\text{+3.239}\times \text{[}{\mathrm{Ln}(\mathrm{WHtR})]}^{3}\text{+0.319}\times \text{1+0.594}\times \mathrm{Ln}(\mathrm{age})$$

For women:

10 $$\text{NVAI }= \text{ } \frac{1}{\text{1} + {\text{e}}^{-\left[\text{-18.765+}\left(\text{0.058}\times {\text{age}}\right)\text{+}\left(\text{0.14}\times {\text{WC}}\right)\text{+}\left(\text{0.057}\times {\text{MAP}}\right)\text{+}\left(\text{0.004}\times {\text{TG}}\right)\text{+}\left(\text{-0.057}\times {\text{HDL}}\right)\right]}}$$

11 $$\text{CVAI }= \text{ } \text{-187.32+}\left(\text{1.71}\times {\text{age}}\right)\text{+}\left(\text{4.23}\times {\text{BMI}}\right)\text{+}\left(\text{1.12}\times {\text{WC}}\right)\text{+}\left(\text{39.76}\times {\text{log}}{\text{TG}}\right)-\left(\text{11.66}\times {\text{HDL}}\right)$$

12 $$\text{VAI }\text{=}\frac{\text{WC}}{\text{36.58+}\left(\text{1.89}\times {\text{BMI}}\right)}\times \frac{\text{TG}}{0.81}\times \frac{1.52}{\text{HDL}}$$

13 $$\text{LAP }\text{=}\left(\text{WC-58}\right)\times {\text{TG}}$$

14 $$\mathrm{METS}-\mathrm{VF}=4.466\text{+0.011}\times \text{[Ln(}{\mathrm{METS}-\mathrm{IR})]}^{3}\text{+3.239}\times \text{[}{\mathrm{Ln}(\mathrm{WHtR})]}^{3}\text{+0.319}\times \text{0+0.594}\times \mathrm{Ln}\left(\mathrm{age}\right)$$

#### Subclinical renal damage definition

Using the estimated glomerular filtration rate (eGFR) as the sole index to determine SRD has limitations. Thus, the urinary albumin creatinine ratio (uACR) was used to jointly reflect the actual renal function of the subjects [27]. Based on the modified calculation formula for Chinese individuals, eGFR = 175 × serum creatinine^−1.234^ × age^−0.179^ (for men). The value needs to be additionally multiplied by 0.79 for women. In the above equation, the units of serum creatinine concentration, age, and eGFR were mg/dl, years, and ml/min/1.73 m^2^, respectively [28, 29]. uACR in mg/mmol was computed by dividing albumin by creatinine based on the urine samples. Participants whose eGFR values fluctuated from 30 to 60 were defined as patients suffering from SRD [30]. Men with uACR values ≥ 2.5 and women with uACR values ≥ 3.5 were also included in this category [31].

#### Statistical analysis

Normally distributed variables were given as the mean ± standard deviation, while other continuous variables were expressed as the median and interquartile range. The number and proportion were used to describe the categorical variables. Based on NVAI tertiles, the overall population was separated into three groups. When comparing parameters among the three groups, the Jonckheere-Terpstra test or the chi-square test was performed where suitable. Spearman correlation analyses revealed the relationships between the obesity indices and the renal function indicators. To examine the connection between NVAI and SRD, binary logistic regression analyses were carried out. Three models were adopted to validate the association, the strength of which was illustrated by the odds ratio (OR). The predictive power of the obesity markers for SRD was proven through receiver operating characteristic (ROC) curves. The area under the curve (AUC), net reclassification index (NRI), and integrated discrimination improvement (IDI) were used to compare the predictive power of the eight adiposity parameters for SRD. All analyses were conducted by SPSS (version 20.0, SPSS Inc., Chicago, IL, USA), Medcalc (version 20.0, Medcalc Ltd., Ostend, Belgium), and R (version 4.2.2, The R Foundation, Vienna, Austria). Two-tailed *P* values of less than 0.05 were considered statistically significant.

## Results

### Characteristics of the NVAI tertiles

Among 2358 participants, their median age was 42.00 years and 1302 were men (55.22%). The whole population was classified into three groups based on NVAI tertiles (Tertile 1, ≤ 0.56; Tertile 2, 0.56–0.90; Tertile 3, > 0.90). The highest tertile group was the highest for age, men’s proportion, incidence of smoking and alcohol drinking, blood pressure, heart rate, FPG, TC, TG, LDL, TBA, GPT, GOT, serum UA, and values of obesity indices. The proportion of patients who had hypertension or diabetes was also found to be notably higher in the top tertile group than in the other groups. The level of uACR increased significantly as the NVAI tertiles increased, while eGFR decreased gradually. In addition, the higher the NVAI values were, the higher the prevalence of SRD (Table 1).

**Table 1:** Characteristics of participants with different NVAI levels

Characteristics	Total (*n* = 2358)	Tertile 1(≤ 0.56)	Tertile 2(0.56–0.90)	Tertile 3(> 0.90)	*P* value
Male [n (%)]	1302(55.22)	155(19.72)	463(58.98)	684(86.91)	< 0.001
Age (years)	42.00(39.00–44.00)	41.00(39.00–44.00)	42.00(39.00–44.00)	43.00(40.00–45.00)	< 0.001
BMI (kg/m^2^)	23.84(21.85–26.00)	21.72 ± 2.03	23.99 ± 2.32	26.29(24.56–28.25)	< 0.001
WC (cm)	84.40(78.00–91.40)	76.38 ± 5.19	85.16 ± 6.15	93.30 ± 7.77	< 0.001
Smoking [n (%)]	1013(42.96)	134(17.05)	347(44.20)	532(67.60)	< 0.001
Alcohol drinking [n (%)]	684(29.01)	85(10.81)	230(29.30)	369(46.89)	< 0.001
Hypertension [n (%)]	482(20.44)	21(2.67)	81(10.32)	380(48.28)	< 0.001
Diabetes mellitus [n (%)]	98(4.16)	12(1.53)	30(3.82)	56(7.12)	< 0.001
SBP (mmHg)	121.33(112.33–131.33)	110.67(104.00–117.33)	120.33(115.00–127.00)	133.00(126.00–143.67)	< 0.001
DBP (mmHg)	75.83(69.00–84.00)	68.00(63.33–73.08)	75.00(70.33–80.00)	85.33(80.33–91.33)	< 0.001
Heart rate (bpm)	73.00(66.00–80.00)	72.50(66.00–80.00)	72.00(66.00–78.00)	75.00(68.00–82.00)	0.001
FPG (mmol/l)	4.57(4.28–4.91)	4.47(4.20–4.76)	4.55(4.28–4.89)	4.73(4.39–5.10)	< 0.001
TC (mmol/l)	4.51(4.04–5.03)	4.43(3.94–4.89)	4.53 ± 0.77	4.63(4.14–5.17)	< 0.001
TG (mmol/l)	1.33(0.96–1.95)	1.05(0.80–1.37)	1.34(0.99–1.95)	1.71(1.25–2.45)	< 0.001
LDL (mmol/l)	2.50(2.14–2.91)	2.41(2.03–2.75)	2.51(2.12–2.91)	2.60(2.27–3.07)	< 0.001
HDL (mmol/l)	1.15(0.99–1.33)	1.29(1.12–1.49)	1.13(0.99–1.30)	1.05(0.92–1.18)	< 0.001
TBA (umol/l)	11.93(9.27–15.55)	11.02(8.54–14.20)	11.89(9.31–15.56)	12.88(9.96–16.45)	< 0.001
GPT (U/l)	19.00(13.00–27.00)	14.00(11.00–19.00)	18.00(14.00–26.00)	25.00(19.00–35.00)	< 0.001
GOT (U/l)	16.00(13.00–20.00)	15.00(12.00–18.00)	16.00(13.00–20.00)	18.00(15.00–23.00)	< 0.001
Serum UA (umol/l)	278.80(225.00–335.18)	229.20(197.98–273.03)	285.79 ± 70.04	327.90(280.20–375.40)	< 0.001
Urine UA (umol/l)	1300.00(927.00–1983.50)	1265.50(871.50–1867.00)	1311.00(948.00–2026.50)	1311.00(957.00–2057.00)	0.004
CVAI	79.94(53.92–111.83)	49.99 ± 20.47	82.11 ± 26.19	118.87(100.20–140.54)	< 0.001
VAI	1.80(1.17–2.81)	1.37(0.95–2.04)	1.82(1.21–2.85)	2.30(1.54–3.51)	< 0.001
LAP	29.82(17.55–50.88)	17.93(11.56–25.85)	30.87(19.84–48.31)	49.91(33.22–77.96)	< 0.001
METS-VF	6.46(6.02–6.81)	5.92(5.64–6.17)	6.49(6.24–6.70)	6.89(6.68–7.09)	< 0.001
ABSI	0.08 ± 0.004	0.08 ± 0.003	0.08 ± 0.003	0.08 ± 0.003	< 0.001
eGFR (ml/min/1.73 m^2^)	97.24(87.16–110.15)	99.06(88.39–111.64)	98.08(87.17–111.11)	95.04(85.31–107.38)	< 0.001
uACR (mg/mmol)	0.98(0.64–1.72)	0.89(0.58–1.40)	0.98(0.64–1.70)	1.13(0.70–2.25)	< 0.001
SRD [n (%)]	315(13.36)	57(7.25)	88(11.21)	170(21.60)	< 0.001

Values are expressed as mean ± standard deviation or n (%)

*ABSI* A body shape index, *BMI* Body mass index, *CVAI* Chinese visceral adiposity index, *DBP* Diastolic blood pressure, *eGFR* estimated glomerular filtration rate, *FPG* Fasting plasma glucose, *GOT* Glutamic oxaloacetic transaminase, *GPT* Glutamic pyruvic transaminase, *HDL* High-density lipoprotein, *LAP* Lipid accumulation product, *LDL* Low-density lipoprotein, *METS-VF* Metabolic score for visceral fat, *NVAI* New visceral adiposity index, *SBP* Systolic blood pressure, *SRD* Subclinical renal damage, *TBA* Total bile acid, *TC* Total cholesterol, *TG* Triglycerides, *UA* Uric acid, *uACR* urinary albumin creatinine ratio, *VAI* Visceral adiposity index, *WC* Waist circumference

### Association of obesity indicators with markers of renal function

Spearman correlation tests were performed to examine the associations between different obesity indices and markers to evaluate the function of the kidney. The results were shown in Table 2. It was suggested that obesity indices except for ABSI were all negatively linked with eGFR but positively correlated with uACR. In brief, the higher the obesity index score, the worse the kidney function.

**Table 2:** Relationships between obesity indices and renal function indicators through Spearman correlation analyses

Obesity indices	eGFR			uACR		
	*r*_s_	95% CI	*P* value	*r*_s_	95% CI	*P* value
BMI	-0.067	-0.108, -0.023	0.001	0.181	0.142, 0.220	< 0.001
WC	-0.053	-0.094, -0.012	0.011	0.145	0.105, 0.183	< 0.001
VAI	-0.093	-0.131, -0.053	< 0.001	0.129	0.090, 0.167	< 0.001
CVAI	-0.103	-0.144, -0.063	< 0.001	0.129	0.092, 0.168	< 0.001
NVAI	-0.094	-0.133, -0.055	< 0.001	0.174	0.135, 0.214	< 0.001
LAP	-0.094	-0.133, -0.053	< 0.001	0.174	0.136, 0.215	< 0.001
METS-VF	-0.061	-0.103, -0.020	0.003	0.161	0.124, 0.201	< 0.001
ABSI	0.041	0.000, 0.079	0.046	0.084	0.045, 0.123	< 0.001

*ABSI* A body shape index, *BMI* Body mass index, *CVAI* Chinese visceral adiposity index, *eGFR* estimated glomerular filtration rate, *LAP* Lipid accumulation product, *METS-VF* Metabolic score for visceral fat, *NVAI* New visceral adiposity index, *uACR* urinary albumin creatinine ratio, *VAI* Visceral adiposity index, *WC* Waist circumference

### Relationship between NVAI and SRD

To investigate the association between NVAI and SRD further, binary logistic regression analyses were conducted. Table 3 demonstrated the relevant results. Generally, the higher the NVAI score was, the greater the risk of SRD in the population. Even after controlling for related confounding variables, including sex, smoking and drinking status, diabetes mellitus, heart rate, TBA, GPT, GOT, TC, LDL, serum UA, and urine UA, a high level of NVAI was still a risk factor for SRD. The OR (95% CI) and variance inflation factor of independent viables in Model 3 were shown in Supplementary Table 1, and no multicollinearity was observed among variables. When choosing the lowest tertile group as a reference, the middle and the top tertile groups of the NVAI demonstrated strong associations with SRD. The ORs of the two groups were 1.920 (95% CI: 1.322, 2.787) and 4.129 (95% CI: 2.750, 6.202), respectively.

**Table 3:** Association between the NVAI and SRD

Tertiles of NVAI	Model 1		Model 2		Model 3	
	OR (95% CI)	*P* value	OR (95% CI)	*P* value	OR (95% CI)	*P* value
Tertile 1 (≤ 0.56)	1	-	1	-	1	-
Tertile 2 (0.56–0.90)	1.615 (1.139, 2.289)	0.007	1.936 (1.341, 2.796)	< 0.001	1.920 (1.322, 2.787)	0.001
Tertile 3 (> 0.90)	3.524 (2.562, 4.846)	< 0.001	4.862 (3.306, 7.152)	< 0.001	4.129 (2.750, 6.202)	< 0.001

Model 1, unadjusted

Model 2, adjusted for sex, smoking status, drinking status and diabetes mellitus

Model 3, adjusted for factors in model 2 plus heart rate, TBA, GPT, GOT, TC, LDL, serum UA and urine UA

*CI* Confidence interval, *GOT* Glutamic oxaloacetic transaminase, *GPT* Glutamic pyruvic transaminase, *LDL* Low-density lipoprotein, *OR* Odds ratio, *TBA* Total bile acid, *TC* Total cholesterol, *UA* Uric acid

### Predictive power of the eight obesity indicators for SRD

**Fig. 2:**
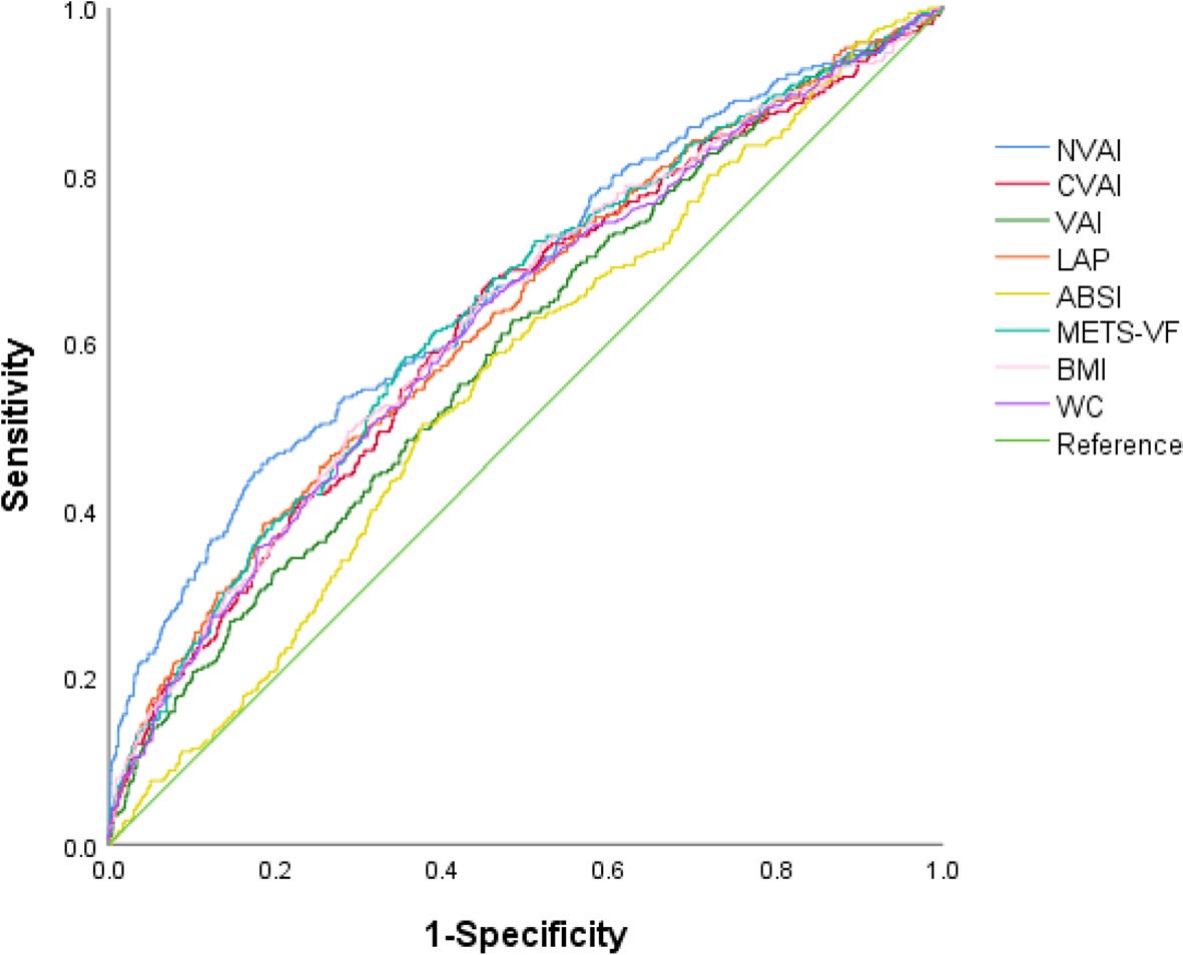
Receiver operating characteristic curves of the NVAI and traditional obesity markers for SRD. Legends of Fig. 2: ABSI, a body shape index; BMI, body mass index; CVAI, Chinese visceral adiposity index; LAP, lipid accumulation product; METS-VF, metabolic score for visceral fat; NVAI, new visceral adiposity index; VAI, visceral adiposity index; WC, waist circumference

The predictive ability of the different obesity indices for SRD was evaluated through ROC curve analyses, and *P* values were obtained by the comparison of the AUCs between the NVAI and the other obesity indicators. As predicted, it was observed that the AUC of the NVAI was 0.666 (95% CI: 0.647, 0.685), which was the largest among all of the obesity indices (Fig. 2, Table 4). Moreover, the incremental predictive values for SRD of different obesity indicators were shown in Table 5. The NRI and IDI of the basic model were significantly enhanced by adding NVAI, CVAI, BMI, WC, LAP, and METS-VF. Among the eight obesity indices, NVAI had the highest NRI (0.392; 95% CI: 0.280, 0.503), and its IDI (0.021; 95% CI: 0.014, 0.027) was second only to that of BMI (0.023; 95% CI: 0.014, 0.032).

**Table 4:** Area under the receiver operating characteristic curves of eight obesity indices for SRD

Obesity indices	AUC	95% CI	*P* value*	Cutoff point	Sensitivity	Specificity
NVAI	0.666	0.647, 0.685	-	0.95	45.40	81.64
CVAI	0.624	0.604, 0.644	< 0.001	82.25	66.35	55.16
VAI	0.594	0.574, 0.614	< 0.001	1.79	62.54	51.59
LAP	0.631	0.611, 0.651	0.016	55.75	38.41	81.40
BMI	0.630	0.611, 0.650	0.006	24.01	65.40	55.46
WC	0.624	0.604, 0.644	< 0.001	85.10	64.13	55.31
ABSI	0.558	0.537, 0.578	< 0.001	0.08	50.16	62.46
METS-VF	0.638	0.618, 0.658	0.005	6.63	58.10	64.46

*ABSI* A body shape index, *AUC* Area under the curve, *BMI* Body mass index, *CI* Confidence interval, *CVAI* Chinese visceral adiposity index, *LAP* Lipid accumulation product, *METS-VF* Metabolic score for visceral fat, *NVAI* New visceral adiposity index, *SRD* Subclinical renal damage, *VAI* Visceral adiposity index, *WC* Waist circumference

^*^Comparison of AUC of NVAI with other obesity indices

**Table 5:** Improvement in prediction for SRD after adding obesity indices

Obesity indices	NRI (95% CI)	*P* value	IDI (95% CI)	*P* value
Basic model	-	*-*	-	*-*
+ NVAI	0.392 (0.280, 0.503)	< 0.001	0.021 (0.014, 0.027)	< 0.001
+ CVAI	0.239 (0.121, 0.357)	< 0.001	0.015 (0.008, 0.022)	< 0.001
+ VAI	0.035 (-0.084, 0.153)	0.569	0.001 (-0.001, 0.002)	0.233
+ LAP	0.257 (0.140, 0.375)	< 0.001	0.010 (0.004, 0.015)	< 0.001
+ BMI	0.307 (0.189, 0.424)	< 0.001	0.023 (0.014, 0.032)	< 0.001
+ WC	0.240 (0.122, 0.358)	< 0.001	0.016 (0.009, 0.023)	< 0.001
+ ABSI	0.087 (-0.032, 0.205)	0.151	0.001 (-0.001, 0.002)	0.298
+ METS-VF	0.349 (0.234, 0.463)	< 0.001	0.017 (0.011, 0.023)	< 0.001

The basic model included sex, smoking status, drinking status, diabetes mellitus, heart rate, TBA, GPT, GOT, TC, LDL, serum UA and urine UA

*ABSI* A body shape index, *BMI* Body mass index, *CVAI* Chinese visceral adiposity index, *IDI* Integrated discrimination improvement, *LAP* Lipid accumulation product, *METS-VF* Metabolic score for visceral fat, *NRI* Net reclassification index, *NVAI* New visceral adiposity index, *SRD* Subclinical renal damage, *VAI* Visceral adiposity index, *WC* Waist circumference

## Discussion

The results of the present study suggested that the higher the NVAI level, the worse the renal function. After correction for various covariates, a high NVAI remained a risk factor for SRD. When compared with other obesity indicators, the NVAI demonstrated greater predictive ability for SRD in Chinese adults.

It is important to find warning parameters of CKD due to its insidious onset and poor prognosis. Obesity, especially visceral obesity, has been revealed to be strongly linked to CKD [7–10]. In a population without obvious cardiovascular diseases, the pararenal fat tissue of patients whose renal function deteriorated dramatically was much thicker than that of subjects in the first and second stages of CKD. The accumulation of pararenal fat was a risk factor for renal function decline [32]. Olivero et al. found that the visceral adipose tissue ratio at baseline had the ability to predict the worsening of CKD stages during the 12 months after renal surgery [33].

Previous research has revealed that indicators to evaluate visceral fat are closely related to CKD. A Chinese study suggested that the level of VAI was significantly associated with urine protein excretion among middle-aged and elderly individuals [13]. Another cross-sectional study proposed that LAP was an independent predictor of CKD in hypertensive patients [34]. The CVAI was also reported to be significantly correlated with a greater prevalence of diabetic kidney disease [21]. Similar results were obtained in this study. A range of obesity indicators all correlated with uACR positively and eGFR negatively. Renal function subsequently deteriorated when the NVAI level increased.

The value of the NVAI as a predictor for SRD was significantly higher than that of the other obesity indices. Possible causes are as follows. BMI dose not reflects the fat distribution or even the total adipose tissue level well because it is influenced by muscle mass [12]. It has been documented that all-cause mortality increased when the BMI score was either too low or too high [35]. Many CKD patients in the later stages often suffer from chronic consumption; thus, relying only on BMI to evaluate body fat may lead to the “obesity paradox” [36, 37]. In addition, volume overload accompanying CKD can also interfere with the assessment ability of BMI. WC is a common indicator used to reflect abdominal fat accumulation, but it is unable to distinguish subcutaneous adipose tissue and visceral adipose tissue [38]. ABSI was developed to assess the risk of obesity-related diseases independently of BMI [17], but was reported to be not superior to BMI or WC in reflecting body fat distribution, either total fat or visceral fat [39].

Based on surveys carried out in the United States, researchers developed LAP to reflect lipid overaccumulation. In Caucasians, the buildup of visceral tissue can be accurately reflected by the VAI. However, both indexes may not be suitable for the Asian population because body fat distribution differs among ethnic groups [40]. The CVAI was established to assess visceral fat distribution in Asians and was reported to outperform the VAI in predicting renal injury [15, 41]. As a composite index combining age, sex, height, weight, WC, glucose, and lipids, METS-VF was considered an excellent predictor of CKD [42]. In this study, we were delighted to observe that the NVAI had a stronger predictive power than the CVAI and the METS-VF for SRD in the Chinese population. The NVAI was initially developed and validated in the Korean population and demonstrated high agreement with computed tomography in assessing visceral obesity. A positive relationship was noted between it and atherosclerotic cardiovascular disease risk[24]. Recently, a Korean study also found that the NVAI had the highest predictive value for coronary atherosclerosis and arterial stiffness among five obesity indices [25]. This study introduced a new perspective by extending the research on the correlation between the NVAI and clinical diseases from the cardiovascular field to the renal disease field.

The underlying causes linking obesity to SRD have been uncovered gradually. First, excess adipose tissue located in and around the kidneys mechanically compresses the renal parenchyma and vessels, which results in increased NaCl resorption in the Henle loop and decreased NaCl delivery to the macula densa. Macula densa feedback maintains the sodium balance by increasing glomerular filtration and activating the renin–angiotensin–aldosterone system. Although glomerular hyperfiltration is a compensatory regulation mechanism of the kidney, a continuously elevated glomerular hydrostatic pressure will eventually cause kidney injury [43]. Second, adipose tissue overload leads to ectopic lipid accumulation [44]. Circulating lipids can accumulate in almost all types of renal cells and generate toxic effects [45, 46]. Excessive accumulation of lipids not only accelerates tubular damage and interstitial fibrosis but also contributes to the development of glomerulosclerosis [45]. Third, the impact of adipokines on the decline of renal function cannot be overstated. Adipocytes produce numerous vital adipokines. For example, interleukin 6, leptin and tumour necrosis factor α take part in not only insulin resistance but also inflammation and other physiological processes [47]. All of these pathogenic changes contribute to accelerating the progression of CKD.

The present research investigated the association between NVAI and SRD for the first time. In addition, it compared the predictive value of NVAI and common obesity indices from different perspectives, which could help identify better predictors in the specific population.

However, some limitations of the current study must be acknowledged. First, it was a single-centre study performed in northern China, whose findings need to be further validated in other ethnic groups. Second, because the majority of participants were middle-aged, caution is advised when extending the conclusions to other age groups. Finally, it is difficult to establish the causality between the NVAI and SRD based on this cross-sectional study. This cohort will continue to be followed up in the future to explore the relationship between dynamic changes in the NVAI and CKD progression.

## Conclusions

In short, the NVAI was proven to show a positive association with SRD. Among eight obesity indicators (BMI, WC, VAI, CVAI, NVAI, and LAP), the NVAI was the best predictor for SRD in the Chinese population. These findings highlight the significance of greater attention being paid to CKD in the population with visceral obesity. NVAI could serve as a reliable marker to recognize obese individuals with an enhanced risk of SRD and facilitate their early diagnosis and treatment, thus avoiding adverse clinical outcomes.

## Supplementary Information

**Additional file 1.**

## Data Availability

The relevant data can be reasonably requested from the corresponding author.

## Abbreviations

ABSI: A body shape index
AUC: Area under the curve
BMI: Body mass index
CI: Confidence interval
CKD: Chronic kidney disease
CVAI: Chinese visceral adiposity index
DBP: Diastolic blood pressure
eGFR: Estimated glomerular filtration rate
FPG: Fasting plasma glucose
GOT: Glutamic oxaloacetic transaminase
GPT: Glutamic pyruvic transaminase
HDL: High-density lipoprotein
IDI: Integrated discrimination improvement
LAP: Lipid accumulation product
LDL: Low-density lipoprotein
MAP: Mean arterial pressure
METS-IR: Metabolic score for insulin resistance
METS-VF: Metabolic score for visceral fat
NVAI: New visceral adiposity index
NRI: Net reclassification index
OR: Odds ratio
ROC: Receiver operating characteristic curve
SBP: Systolic blood pressure
SRD: Subclinical renal damage
TBA: Total bile acid
TC: Total cholesterol
TG: Triglyceride
UA: Uric acid
uACR: Urinary albumin creatinine ratio
VAI: Visceral adiposity index
WC: Waist circumference
WHtR: Waist to height ratio
